# Supplementary material for: Vitamin D Modified DSS-Induced Colitis in Mice via STING Signaling Pathway
Source: Biology (Basel). 2025 Jun 18;14(6):715. doi: 10.3390/biology14060715 (PMC12190092; doi:10.3390/biology14060715)
Supplement: Supplementary file 1 [file biology-14-00715-s001.zip › Supplementary Materials S1: 16s-report of gut microbiota/Groups/diff_analysis/UnivarTestMETACYC/figure/barplot/top10log.pdf]

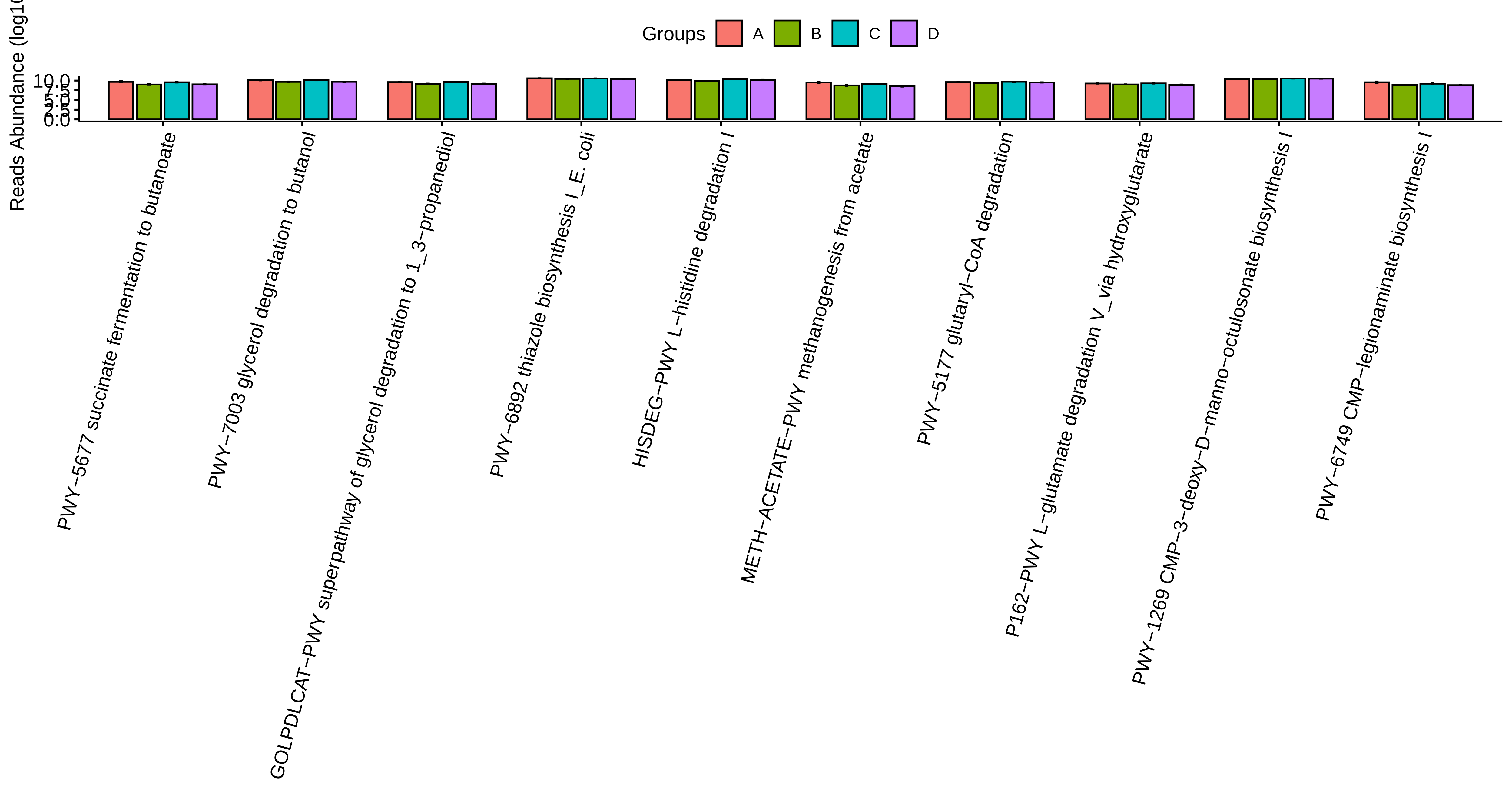

Groups
A
B
C
D
Reads Abundance (log10
10.0
7.5
5.0
2.5
0.0
PWY−5677 succinate fermentation to butanoate
PWY−7003 glycerol degradation to butanol
GOLPDLCAT−PWY superpathway of glycerol degradation to 1_3−propanediol
PWY−6892 thiazole biosynthesis I_E. coli
HISDEG−PWY L−histidine degradation I
METH−ACETATE−PWY methanogenesis from acetate
PWY−5177 glutaryl−CoA degradation
P162−PWY L−glutamate degradation V_via hydroxyglutarate
PWY−1269 CMP−3−deoxy−D−manno−octulosonate biosynthesis I
PWY−6749 CMP−legionaminate biosynthesis I
